# Biomolecular condensates in lung cancer: from molecular mechanisms to therapeutic targeting

**DOI:** 10.1038/s41420-025-02735-y

**Published:** 2025-10-06

**Authors:** Nannan Wang, Qianqian Liu, Lingrui Shang, Tongran Zhang, Tao Xu, Shangbang Gao, Lihua Chen, Huisheng Liu

**Affiliations:** 1 College of Life Science and Technology, Huazhong University of Science and Technology, Wuhan, Hubei China; 2 Guangzhou National Laboratory, Guangzhou, Guangdong China; 3 School of Biomedical Engineering, Guangzhou Medical University, Guangzhou, Guangdong China; 4 Shenzhen University Medical School, Shenzhen, China; 5 School of Forestry and Biotechnology, Zhejiang A&F University, Hangzhou, China; 6 National Key Laboratory for Development and Utilization of Forest Food Resources, Zhejiang A&F University, Hangzhou, China

**Keywords:** Lung cancer, Business strategy in drug development

## Abstract

Lung cancer constitutes a globally prevalent malignancy with high morbidity and mortality, imposing a substantial burden on public health systems worldwide. Growing evidence indicates that the initiation and progression of lung cancer involve multiple biological processes. Liquid-liquid phase separation (LLPS), a fundamental mechanism orchestrating diverse cellular biochemical events, has been increasingly implicated in lung cancer pathogenesis, particularly in tumorigenesis and chemoresistance. These findings unveil promising opportunities for pharmacological intervention through condensate-targeting therapeutics. Herein, we review the composition, regulatory mechanisms, and functional roles of biomolecular condensates in lung cancer progression. We further explore their potential applications in diagnosis, therapeutic strategies, and drug development, while addressing the current challenges and future research directions in this field. Elucidating the mechanistic interplay between phase separation and lung carcinogenesis holds significant promise for advancing novel therapeutic avenues in precision oncology.

## Facts

- Lung cancer continues to be one of the leading causes of cancer incidence and mortality worldwide, yet the clinical need for treatment remains largely unmet.
- LLPS plays a key role in the modulation of bioprocesses and drug metabolism in cancer cells, making it a promising therapeutic target and diagnostic biomarker.
- LLPS has emerged as a critical regulatory mechanism underlying lung cancer progression.

## Open questions

- Does LLPS contribute to oncogenesis and modulation of drug sensitivity in lung cancer cells?
- Can LLPS be leveraged as a potential target for therapeutic intervention in lung cancer?

## Introduction

Lung cancer, which is a malignant tumor that originates in the bronchial epithelium, has shown a progressive increase in both incidence and mortality rates, posing a significant global health burden [1]. Characterized by high tumor heterogeneity and insidious early-stage progression, lung cancer often results in late-stage diagnosis. Histologically, lung cancer is divided into two main types: small cell lung cancer (SCLC) and non-small cell lung cancer (NSCLC). Approximately 85% of NSCLC cases are classified as lung adenocarcinoma (LUAD) or lung squamous cell carcinoma (LUSC). These two distinct types have unique molecular profiles and clinical behaviors that dictate therapeutic approaches. NSCLC is typically managed through surgery, chemotherapy, radiotherapy, targeted therapy, immunotherapy, or multimodal approaches, whereas SCLC predominantly receives combined radiotherapy and chemotherapy. However, the five-year survival rate remains suboptimal, primarily due to the rapid progression of the disease and high rates of recurrence after treatments.

In eukaryotic cells, biomolecules initiate LLPS upon exceeding a critical concentration threshold, leading to their aggregation into a dense phase [2, 3]. This nascent condensate subsequently recruits additional proteins and nucleic acids through specific multivalent interactions or functional complementarity, maturing into a phase-separated condensate [4, 5]. Compared to conventional membrane-bound organelles, these condensates exhibit enhanced spatiotemporal flexibility due to the dynamic exchange of their molecular components, subcellular localization, and functional states [6, 7]. This inherent dynamism enables condensates to precisely regulate intracellular biochemical reactions, controlling their initiation and termination in response to cellular demands.

LLPS has emerged as a critical regulatory mechanism underlying lung cancer progression. Biomolecular condensates formed via LLPS, including Cajal bodies [8], stress granules (SGs) [9], and nuclear speckles [10], are implicated in tumor pathogenesis. Notably, LLPS is linked to therapeutic resistance and poor prognosis in lung cancer [11–14]. Consequently, studying LLPS represents a promising framework for elucidating tumor biology and developing anti-cancer therapeutics. This review first summarizes the composition and regulatory mechanisms of LLPS. Subsequently, it details the roles of LLPS in lung cancer progression. Finally, the potential implications of LLPS for lung cancer diagnosis, drug discovery, and prognosis are discussed. A deeper understanding of the pathogenic functions of LLPS in lung cancer may facilitate the development of innovative therapeutic strategies for managing this malignancy.

## Liquid-Liquid Phase Separation

Phase-separated condensates have been referred to as liquid droplets, puncta, membraneless organelles, and biomolecular condensates [2, 6]. These structures are localized to both nuclear and cytoplasmic compartments and are involved in regulating diverse fundamental cellular processes.

### Composition of condensates

**Fig. 1:**
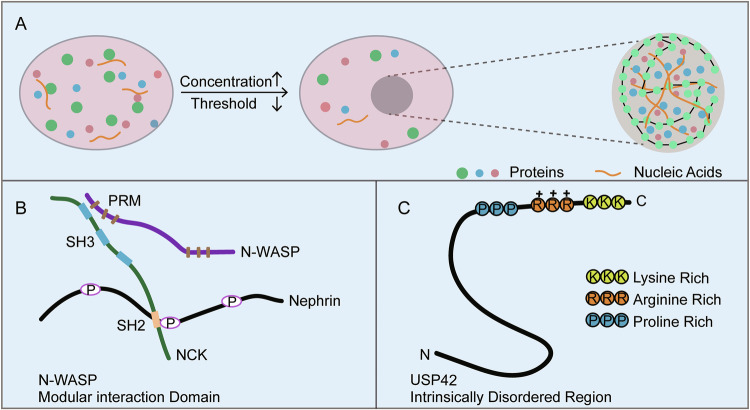
Biomolecular condensate formation and intermolecular interaction patterns. **A** Phase separation schematic depicting membraneless organelle formation when intracellular protein/nucleic acid concentrations exceed saturation thresholds, resulting in partitioning into dense and dilute phases. **B** Molecular architecture of NCK-mediated condensate assembly. SH2 domain specifically binds phosphorylated tyrosine motifs on Nephrin, while SH3 domain interacts with proline-rich motifs (PRM) on N-WASP. These multivalent interactions drive the formation of high order oligomer that reduce solubility and promote phase separation. **C** Structural determinant analysis of USP42. The C-terminal IDR containing consecutive proline-rich, arginine-rich, and lysine-rich motifs constitutes the critical domain for phase separation capability. The authors confirm that all figures and graphical abstract were created by the authors themselves without the use of any third-party software or paid services.

The Hyman Laboratory first documented LLPS in eukaryotic cells in 2009 through their investigation of germ cells in *Caenorhabditis elegans* [2]. Brangwynne demonstrated that P granules undergo posterior aggregation within germ cells, thereby triggering intracellular phase transitions that ultimately promote cell division. Physically, P granules exhibit liquid-phase properties such as fusion dynamics, droplet formation, surface wetting, and the ability to undergo spontaneous or force-induced dissolution and condensation. Further studies of nucleoli and chromatin reveals that condensates are composed principally of nucleic acids (DNA and RNA) and proteins (Fig. 1A), with sizes ranging from the nanometer to micrometer scale [15, 16]. Structurally, condensate proteins can be classified into two primary categories: scaffold proteins and client proteins [17]. Scaffold proteins predominantly localize to the peripheral regions of condensates, where they offer structural integrity and facilitate material exchange. Within the condensate interior, client proteins and nucleic acid components engage with scaffold proteins through intermolecular interactions. Furthermore, condensate composition is dynamic rather than static. The biological macromolecules within these condensates are continuously moving and actively exchanging materials with their surroundings.

### Mechanisms of LLPS condensate formation

Condensates formation is driven by multivalent interactions between proteins and nucleic acids [5]. These interactions, occurring both intermolecularly and intramolecularly, commonly involve modular interaction domains or intrinsically disordered regions (IDRs). These structural features facilitate multivalent binding, drive the assembly of complexes, and lead to reduced solubility of biomolecules in solution [18]. For instance, the actin-regulatory complex comprising Nephrin, NCK, and neural Wiskott-Aldrich syndrome protein (N-WASP) undergoes phase separation upon significant activation of Arp2/3, an actin nucleation factor. The assembly of this N-WASP complex exemplifies how multivalent interactions orchestrate protein condensation and regulate phase transitions [18] (Fig. 1B). IDRs are typically enriched in glycine (G), serine (S), glutamine (Q), arginine (R), lysine (K), and proline (P), promoting the formation of flexible structural motifs [19, 20]. Furthermore, charged and aromatic residues also play critical roles in LLPS [21–24]. These amino acids modulate protein charge distribution, enabling cation-π and π-π interactions that enhance binding affinity between proteins and nucleic acids [23, 25]. For example, the human germ granule protein DEAD-box helicase 4 (DDX4) spontaneously undergoes LLPS both in vivo and in vitro, driven by electrostatic complementarity. Therefore, disruption of DDX4 charge patterning effectively dissolves its condensates [26]. Similarly, ubiquitin-specific peptidase 42 (USP42) LLPS is primarily mediated by a proline-, arginine-, and lysine-rich (PRK) domain located at its C-terminus. In contrast, the N-terminal region of USP42 forms insoluble aggregates in vitro and does not undergo LLPS [10] (Fig. 1C).

The propensity for proteins to undergo LLPS is further influenced by environmental factors, including temperature, pH, ionic strength, and macromolecular crowding agents. These factors primarily influence multivalent interactions underpinning condensate formation, such as π–π stacking, cation–π interactions, electrostatic forces, hydrophobic interactions, and hydrogen bonding. For instance, the Bub3-interacting and GLEBS-containing protein Z (BuGZ) undergoes LLPS, predominantly driven by hydrophobic interactions, exhibiting pronounced temperature sensitivity [21]. In contrast, superoxide dismutase 1 (SOD1) and microtubule-associated protein Tau form condensates governed by both electrostatic and hydrophobic forces, rendering them highly sensitive to salt concentrations due to electrostatic shielding effects [27, 28]. Notably, LLPS in most proteins results from the cooperative modulation of multiple factors. This is exemplified by nuclear ribonucleoprotein A1 (hnRNPA1), whose LLPS is modulated by its low-complexity domain (LCD) and aromatic amino acid residues. Consequently, hnRNPA1 condensate formation is critically dependent on multiple factors, including ionic strength, pH, and temperature [29]. Furthermore, macromolecular crowding agents promote condensate formation by mimicking the intracellular milieu and enhancing local protein concentrations [29–31].

## Phase-Separated Condensates In Lung Cancers

**Fig. 2:**
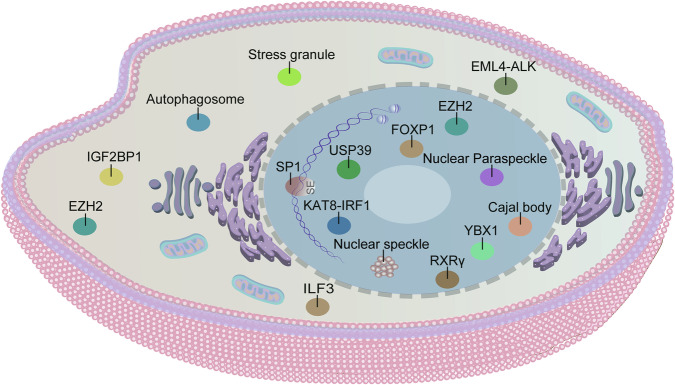
Subcellular localization of experimentally characterized biomolecular condensates associated with lung cancer pathogenesis. Schematic representation of reported biomolecular condensates in lung cancer. These condensates are localized to the cytoplasm, nucleus, and chromatin, participating in diverse cellular processes that regulate tumorigenesis and progression. The authors confirm that all figures and graphical abstract were created by the authors themselves without the use of any third-party software or paid services.

LLPS is a fundamental biological process governing diverse cellular functions in eukaryotic cells. While essential for critical physiological roles in normal cells, dysregulation of LLPS underlies various pathological states, including tumorigenesis. Accumulating evidence indicates that oncogenesis is driven by aberrant gene expression patterns concomitant with dysregulated phase separation. This section examines the mechanisms by which LLPS regulates key cellular processes in lung cancer and the pathological implications of its dysregulation for tumor development (Fig. 2).

### Stress granules

In response to starvation, hypoxia, drug exposure, or viral infection, cells rapidly form SGs to reduce the synthesis of non-essential proteins and adjust their energy metabolism. Many macromolecules dynamically assemble into SGs to coordinate physiological activities including mRNA localization, translation regulation, degradation, antiviral responses, and tumorigenic signaling pathways [32, 33]. Component analyses reveal that SG formation coincides with translational repression. Consequently, SGs contain abundant translation initiation factors, RNA-binding proteins (RBPs), and untranslated mRNAs [34, 35]. SGs are widely recognized as condensates [36–38]. The Ras GTPase-activating protein-binding proteins 1 and 2 (G3BP1 and G3BP2) serve as core scaffold proteins essential for driving SG formation. Expression of either protein alone is sufficient to initiate SG condensation [37].

Accumulating evidence suggests that SGs function as regulatory hubs influencing the progression of lung cancer. Mutation or dysregulation of SG regulators impairs therapeutic efficacy, immune responses, and clinical prognosis in NSCLC [34]. Mechanistically, the tumor suppressor tripartite motif-containing protein 72 (TRIM72, also known as MG53) regulates NSCLC proliferation and migration by modulating G3BP2 activity [9]. Conversely, in NSCLC cells, the oncogene ETS variant transcription factor 4 (*ETV4*) suppresses hexokinase-1 (*HK1*) activity, releases the inhibition of histone deacetylase 6 (HDAC6) and G3BP2 expression, and promotes formation of the lysosomal-TSC2 complex. Collectively, these events attenuate signaling through the mechanistic target of rapamycin complex 1 (mTORC1) and enhance cellular stress adaptation [33]. Furthermore, SGs can regulate SARS-CoV-2 infection mediated by the viral nucleocapsid (N) protein under stress conditions [32].

### Nuclear paraspeckles

Nuclear paraspeckles are membraneless, spherical subnuclear bodies predominantly involved in transcriptional regulation and alternative splicing [39]. Initially identified by Archa in 2002 [40], their core structural components comprise the proteins non-POU domain-containing octamer-binding protein (p54/nrb), splicing factor proline- and glutamine-rich (SFPQ), paraspeckle component 1 (PSPC1), and the essential long non-coding RNA nuclear paraspeckle assembly transcript 1 (NEAT1). NEAT1 specifically localizes to paraspeckles, serving as an essential structural scaffold for their assembly. Paraspeckle formation is directly dependent on NEAT1 expression levels. Furthermore, NEAT1 orchestrates the recruitment and localization of p54/nrb and SFPQ to paraspeckles [41]. In NSCLC, the transcription factor octamer-binding transcription factor 4 (OCT4) binds the *NEAT1* promoter, inducing its upregulation. Elevated NEAT1 expression correlates positively with larger tumor size, advanced clinical stage, increased metastatic potential, vascular invasion, and poor postoperative survival [42]. Notably, a clinical study from northern China showed an association between *NEAT1* polymorphism rs2230905 and an increased risk of lung adenocarcinoma [43].

Mechanistically, NEAT1 functions as a competitive endogenous RNA (ceRNA) by sequestering miR-let-7a. This sequestration relieves let-7a-mediated repression of its downstream target insulin-like growth factor 2 (IGF-2), thereby promoting NSCLC cell proliferation and metastasis [12]. In LUAD, high NEAT1 expression inhibits miR-335 function, leading to derepression of hepatocyte growth factor receptor (c-MET) and contributing to sorafenib resistance [44]. Additionally, epigallocatechin-3-gallate (EGCG) enhances reactive oxygen species (ROS) generation, downregulates ERK1/2 signaling, and upregulates both NEAT1 and the copper transporter 1 (CTR1). This NEAT1 upregulation promotes cisplatin sensitivity [14]. NEAT1 also interacts with DNA methyltransferase 1 (DNMT1) to inhibit the tumor suppressor p53 and suppress cyclic GMP-AMP (cGAMP) synthesis. Consequently, this interaction inhibits the cyclic GMP-AMP synthase–stimulator of interferon genes (cGAS-STING) pathway, facilitating tumor immune evasion from T cell-mediated surveillance [13].

### Autophagosome

Autophagy is a conserved, degradative cellular process that facilitates the recycling of cytoplasmic components. This process enables the clearance of damaged organelles, degradation of macromolecules, and elimination of intracellular pathogens. Accumulating evidence indicates that LLPS serves as a critical regulatory mechanism in autophagy, modulating autophagic flux and influencing cell fate. For instance, calcium transients at the endoplasmic reticulum (ER) surface induce LLPS of focal adhesion kinase family-interacting protein of 200 kDa (FIP200). This phase transition recruits components of the Unc-51-like autophagy activating kinase (ULK) complex, thereby spatially organizing autophagy initiation [45]. Additionally, p62/SQSTM1, a key scaffold protein for autophagosome formation, forms LLPS-driven condensates that concentrate ubiquitinated cargo. These p62/SQSTM1 condensates selectively recruit ubiquitinated substrates through specific non-covalent multivalent interactions and facilitate their subsequent encapsulation into autophagosomes. These findings collectively demonstrate that LLPS orchestrates autophagosome assembly site formation, modulates the kinetics of autophagy initiation, and governs substrate selectivity during autophagic encapsulation, highlighting its pivotal regulatory role in autophagy [46, 47].

The regulation of autophagy is primarily orchestrated by three key signaling pathways: the AMPK/mTOR, PI3K/AKT, and MAPK/ERK1/2 cascades. Agents such as lactoferrin (LTF) and gitogenin induce AMPK phosphorylation, leading to activation of the AMPK/mTOR pathway [48–51]. This activation triggers autophagy, suppresses cell proliferation, and promotes apoptosis and radioresistance. Conversely, activation of the PI3K/AKT/mTOR pathway by factors like microRNA-199a-5p and C-C motif chemokine ligand 2 (CCL2) inhibits autophagy, enhances cell proliferation, migration, and invasion, and reduces both apoptosis and drug resistance [52, 53]. Similarly, activation of the MAPK/ERK1/2/mTOR pathway promotes tumorigenesis by inhibiting autophagy [54].

However, the precise role of autophagy in lung cancer pathogenesis remains contentious. For instance, ubiquitin-specific peptidase 15 (USP15) reportedly induces autophagy via the TNF receptor-associated factor 6 (TRAF6)-Beclin 1 (BECN1) signaling axis, suppressing lung cancer progression [55]. In contrast, fucosyltransferase 2 (FUT2)-mediated induction of autophagy in LUAD inhibits apoptosis [56]. Furthermore, autophagy inhibition may also enhance drug resistance [57]. Additionally, autophagy can degrade the transcription factor SRY-box transcription factor 2 (SOX2), reducing cancer stemness and promoting tumor cell differentiation [58]. Nonetheless, some evidence suggests that autophagy may facilitate the self-renewal of lung cancer stem cells by degrading ubiquitinated p53 [59]. Collectively, these findings highlight that the functional consequences of autophagy are highly context-dependent, varying with the specific activating stimuli and cellular milieu.

### Cajal bodies

Cajal bodies are localized within the nucleoplasm of eukaryotic cells. WD repeat domain 79 (WDR79), a WD-repeat protein and essential scaffolding component for Cajal body assembly, is highly expressed in NSCLC [60–62]. Depletion of WDR79 significantly inhibits NSCLC cell proliferation and induces apoptosis [8].

Mechanistically, WDR79 interacts with ubiquitin-specific protease 7 (USP7), which reduces ubiquitination of the E3 ligase mouse double minute 2 homolog (MDM2) and its substrate p53. This stabilization extends the half-life of both proteins, thereby promoting proliferation [63]. Additionally, WDR79 protects ubiquitin-like protein containing PHD and RING finger domains 1 (UHRF1) against polyubiquitination-mediated degradation. This facilitates UHRF1-dependent DNA methylation and histone modifications, consequently enhancing NSCLC tumorigenesis [64].

### Nuclear speckles

Nuclear speckles compartmentalize diverse mRNA splicing factors and protein processing factors essential for regulating protein synthesis and assembly. The speckle-type POZ protein (SPOP), a major component of nuclear speckles, localizes to these structures. Functioning as a tumor suppressor, SPOP expression is frequently suppressed in NSCLC via methylation of its promoter region [65, 66]. This suppression consequently impairs SPOP-mediated regulation of the NF-κB pathway [67, 68].

Additionally, the deubiquitinating enzyme USP42 localizes to SC-35-positive nuclear speckles in an enzyme activity-dependent manner via its C-terminal positively charged residues. USP42 recruits the alternative splicing component pleiotropic regulator 1 (PLRG1), promoting its LLPS and integration into nuclear speckles. This process modulates mRNA alternative splicing, thereby significantly influencing tumor cell growth [10].

### Other key lung cancer-related proteins

Beyond the previously characterized condensates, cells harbor numerous uncharacterized condensates. Aberrantly expressed proteins or dysregulated nucleic acids can induce the formation of these condensates, ultimately drives tumor progression and confers therapeutic resistance in NSCLC.

Biomolecular condensates play significant roles in the pathogenesis of lung cancer. For instance, the upregulation of lncRNAs MELTF-AS1 and MNX1-AS1 promotes tumorigenesis and progression by inducing phase separation of downstream target proteins [69, 70]. Furthermore, post-translational modifications (PTMs) such as deubiquitination and myristoylation contribute directly or indirectly to condensate formation, facilitating tumor development [71, 72]. Condensates involving echinoderm microtubule-associated protein-like 4-anaplastic lymphoma kinase (EML4-ALK), specificity protein 1 (SP1), and Src homology 2 (SH2) proteins potently activate downstream oncogenic signaling pathways [73–75].

Additionally, condensates contribute to therapeutic resistance mechanisms. The oncogene *c-MYC* activates *MYLK-AS1* transcription, which subsequently promotes interleukin enhancer binding factor 3 (ILF3) phase separation; the resulting ILF3 condensates stabilize glutamate dehydrogenase 1 (*GLUD1*) mRNA, enhancing mitochondrial glutamine metabolism and conferring resistance to tyrosine kinase inhibitors (TKIs) [76]. Similarly, interactions between forkhead box P1 (FOXP1) and the specificity protein 8 super-enhancer (SP8-SE) form transcriptional condensates that upregulate SP8 expression. This promotes homologous recombination repair in SCLC, leading to increased chemoresistance [77]. Likewise, retinoid X receptor gamma (RXRγ) condensates enhance the transcription of target genes, promoting tumor stemness and metastasis, ultimately leading to chemoresistance in SCLC [78].

Biomolecular condensates also modulate anti-tumor immunity. Exposure to Interferon-gamma (IFNγ) induces formation of lysine acetyltransferase 8-interferon regulatory factor 1 (KAT8-IRF1) condensates that bind to the programmed death-ligand 1 (*PD-L1*) promoter, augmenting its transcription and enabling tumor cells to evade immune surveillance [79]. Collectively, condensates exert multifaceted roles in tumor initiation, progression, and therapeutic resistance (Table 1).

**Table 1:** Biomolecules implicated in lung cancer tumorigenesis mediated by LLPS.

Condensate	Biomolecular	Location	Role of phase separation in tumor	Reference
Stress Granule	G3BP1 G3BP2	Cytoplasm	MG53 modulates G3BP2/SG activity to regulate cell proliferation and migration	[9]
			ETV4 promotes lysosomal-TSC2 complex formation, thereby inhibiting mTORC1 and enhancing cellular adaptation to stress	[33]
			Under stress conditions, the SARS-CoV-2 nucleocapsid protein translocates into SGs and participates in condensate formation	[32]
Nuclear Paraspeckle	NEAT1	Nucleus	NEAT1 relieves let-7a-mediated repression of IGF2, thereby promoting cell proliferation and metastasis	[12]
			High expression of NEAT1 regulates cellular sensitivity to cisplatin and sorafenib	[14, 44]
			NEAT1 interacts with DNMT1 to inhibit cyclic GMP-AMP synthase, thereby facilitating tumor immune evasion	[13]
Autophagosome	P62/SQSTM1 Atg1 FIP200	Cytoplasm	Agents activate autophagy through the AMPK/mTOR pathway, suppress cell proliferation, and promote apoptosis and radiosensitivity	[48–51]
			Agents inhibit autophagy through the PI3K/AKT and MAPK/ERK1/2 pathways, thereby promoting tumorigenesis and drug resistance	[52–54]
			USP15 induces autophagy via the TRAF6-Beclin-1 signaling axis, suppressing lung cancer progression	[55]
			FUT2 facilitates autophagy and suppresses apoptosis via p53 and JNK signaling	[56]
			CA3 increases DUSP1 expression and concomitantly induces autophagy, delaying osimertinib resistance	[57]
			Autophagy degrades SOX2, reducing cancer stem cell properties and promoting tumor cell differentiation	[58]
			Autophagy augments the self-renewal capacity of lung cancer stem cells by degrading ubiquitinated p53	[59]
Cajal Body	WDR79	Nucleus	WDR79 decreases ubiquitination of UHRF1, p53, and MDM2, stabilizing their protein levels and thus promoting cell proliferation and tumorigenesis	[63, 64]
Nuclear speckles	USP42 PLRG1	Nucleus	USP42 orchestrates PLRG1 recruitment to nuclear speckles and its colocalization with SC35, thereby driving tumorigenesis	[10]
IGF2BP1	IGF2BP1 MNX1-AS1	Cytoplasm	LncRNA MNX1-AS1 facilitates IGF2BP1 phase separation, promoting cell cycle progression and proliferation	[69]
YBX1	YBX1 MELTF-AS1	Nucleus	SP1 transcriptionally activates lncRNA MELTF-AS1, triggering LLPS of YBX1 and regulating tumorigenesis	[70]
EZH2	EZH2 STAT3	Nucleus	N-terminal glycine myristoylation of EZH2 induces LLPS, activates STAT3 signaling, and enhances cellular proliferation	[71]
USP39	USP39	Nucleus/Cytoplasm	USP39 condensates promote cell proliferation and migration by regulating GLI1 expression	[72]
SP1	SP1	Nucleus	SP1 condensates mediate super-enhancer assembly to upregulate RGS20 expression, driving tumor cell proliferation and metastasis	[74]
EML4-ALK	EML4 ALK	Cytoplasm	The EML4-ALK fusion protein undergoes LLPS, driving hyperactivation of oncogenic signaling through aberrant protein aggregation	[75]
ILF3	ILF3 MYLK-AS1	Cytoplasm	ILF3-MYLK-AS1 condensates accelerate mitochondrial glutamine metabolism and promote TKI resistance	[76]
FOXP1	FOXP1	Nucleus	The FOXP1-SP8-SE transcription complex promotes SP8 transcription and mediates chemoresistance	[77]
RXRγ	RXRγ LSD1	Nucleus	RXRγ-LSD1 condensates promote tumor stemness and metastasis, driving chemotherapy resistance	[78]
KAT8-IRF1	KAT8 IRF1	Nucleus	KAT8-IRF1 condensates upregulate PD-L1 expression to facilitate tumor immune evasion	[79]

## Applications Of Phase Separation In Lung Cancer

As mentioned above, LLPS plays a critical role in the regulation of the onset and progression of lung cancer. This section will review the potential role of condensates in lung cancer diagnosis, treatment, and prognosis, exploring their utility as novel targets for therapeutic intervention and clinical management of the disease.

### Early diagnostics

A significant challenge in the clinical management of lung cancer is the absence of overt symptoms during the early stages, which often results in diagnosis only at advanced stages. Due to their marked heterogeneity and metastatic potential, advanced tumors are often not amenable to surgical intervention. Consequently, the early detection is critical for improving patient survival rates. Despite the absence of definitive evidence supporting the use of phase-separated proteins as biomarkers for lung cancer, several studies have demonstrated their potential.

First, USP42 occurs phase separation in lung cancers, which directs the integration of the spliceosome component PLRG1 into nuclear speckles to influence a set of cancer-related genes such as chromosome 18 (SS18) and large tumor suppressor kinase 1 (LATS1) [10]. The identification of specific genes whose expression is altered due to aberrant LLPS has the potential to serve as biomarkers for early diagnosis.

Second, the tumor suppressor p53 undergoes phase separation under conditions of DNA damage stress. Conversely, p53 oncogenic mutations impair the formation of p53 condensates, resulting in a decrease in the activation of target genes and the promotion of tumorigenesis [80]. Since p53 is one of the most frequently mutated genes in lung cancer [81], it is hypothesized that p53 phase separation property may serve as a pathological diagnostic marker for lung cancer.

Third, the cytosolic Yes-associated protein (YAP) has been demonstrated to modulate the expression of diverse oncogenic genes in NSCLC. Its nuclear translocation and phase-separation enhance transcriptional activity, thereby contributing to tumorigenesis [73]. Therefore, the presence of YAP nuclear condensates may serve as a biomarker for NSCLC.

### Drug discovery

Drug resistance remains significant contributing factor to the failure of cancer treatment regimens. As outlined earlier, biomolecular condensates have emerged as critical contributors to drug resistance mechanisms. This understanding suggests that targeted drug delivery and efficacy modulation could potentially be achieved through precise control of phase separation processes and biophysical properties of condensates. Indeed, recent advances have revealed the tremendous potential of condensate biology in developing innovative therapeutic strategies and next-generation drug delivery platforms.

The transcriptional coactivators bromodomain-containing protein 4 (BRD4) and mediator complex subunit 1 (MED1) form condensates at super-enhancer sites, thereby compartmentalizing and concentrating the transcription apparatus at key cell-identity genes to ensure their robust transcription [30]. Subsequent fluorescent labeling of cisplatin revealed that the labeled compound accumulated in MED1 condensates, exhibiting high-speed dynamics and co-localization with platinum-associated DNA [82]. Furthermore, studies on the estrogen receptor alpha (ERα) mutant protein have demonstrated a ~ 10-fold reduction in its affinity for tamoxifen within MED1 condensates, which correlated with diminished therapeutic efficacy of the drug. The capacity of MED1 and BRD4 to concentrate small-molecule drugs highlights their potential as novel targets for drug delivery systems. In research on castration-resistant prostate cancer (CRPC), the androgen receptor (AR) antagonist enzalutamide was shown to disrupt aggregate formation in wild-type AR. In contrast, it enhanced LLPS in the drug-resistant AR mutant (F877L/T878A), paradoxically amplifying AR signaling. By means of high-throughput screening, researchers identified a small-molecule compound, ET516, that effectively inhibits LLPS in both wild-type and drug-resistant AR mutants [83]. Collectively, these studies illustrate how phase separation dynamically redistributes drug concentrations by modulating condensate formation, thereby improving target engagement efficiency between drugs and their molecular targets.

### Prognostic markers

The identification of robust prognostic markers is critical for lung cancer management, as it enables personalized treatment strategies and improved prediction of clinical outcomes across diverse patient subgroups. As mentioned above indicates that elevated expression of specific molecular features may serve as prognostic biomarkers. For instance, the presence of the EML4-ALK fusion oncoprotein is a well-established biomarker specifically indicative of LUAD [75]. Similarly, high expression of the lncRNA NEAT1 shows an inverse correlation with 5-year survival rates, suggesting its potential utility as a predictor of clinical outcomes [11, 84].

Significantly, integrating bioinformatics analysis of clinical gene expression data with LLPS biology has emerged as a powerful approach for cancer prognosis prediction. For example, integrating LUAD transcriptomic profiles from The Cancer Genome Atlas (TCGA) and Gene Expression Omnibus (GEO) databases with LLPS-associated protein data from DrLLPS and PhaSepDB has yielded novel insights. Differential gene expression (DGE) analysis identified 17 LLPS-related genes among 5445 total differentially expressed genes (DEGs) as prognostic risk factors [85, 86]. This methodology has been successfully applied to other malignancies. For example, Functional enrichment analysis using LLPS-related genes in low-grade glioma established distinct high-risk and low-risk patient subgroups with significantly divergent prognoses [87]. Similarly, in esophageal adenocarcinoma, bioinformatics-based clustering and differential analysis of TCGA data using LLPS-related genes enabled effective risk stratification [88]. The synergistic integration of bioinformatics with LLPS biology presents a promising approach to advance clinical translation, particularly for developing novel diagnostic and therapeutic strategies for cancer.

## Concluding Remarks And Future Perspectives

This review synthesizes recent advances in understanding biomolecular condensates during lung cancer progression. It elucidates how these dynamic assemblies dysregulate oncogenic pathways involving RNAs and proteins, thereby driving pathological LLPS. Critically, emerging evidence indicates that targeted disruption of LLPS may yield novel therapeutic candidates for drug delivery platforms. Although research on LLPS in eukaryotic systems remains in its infancy, necessitating deeper mechanistic insights and functional characterization, it still holds significant potential to revolutionize therapeutic strategies.

**Fig. 3:**
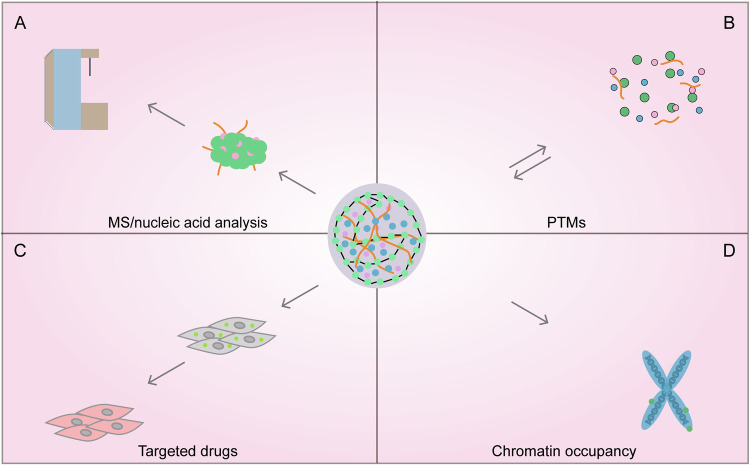
Prospective research directions of LLPS in biomedical sciences. **A** Identifying and validating condensate protein and nucleic acid components across diverse cellular microenvironments. **B** Investigating how post-translational modifications (e.g., phosphorylation, acetylation, SUMOylation) modulate phase separation. **C** Discovering small molecules or peptides capable of regulating pathological condensates. **D** Analyzing and elucidating aberrant condensate-chromatin binding interactions and their underlying regulatory mechanisms. The authors confirm that all figures and graphical abstract were created by the authors themselves without the use of any third-party software or paid services.

Consequently, elucidating LLPS significance in cancer through advanced tools represents a promising frontier. Conventional LLPS studies primarily rely on immunofluorescence staining or optogenetic approaches to characterize phase-separated molecules and delineate their associated signaling pathways. However, these methods are limited in their ability to systematically identify key mediators of these processes. We propose that advancing the field requires addressing the following key objectives: (1) Identifying and validating condensate protein and nucleic acid components across diverse cellular microenvironments; (2) Investigating how post-translational modifications (e.g., phosphorylation, acetylation, SUMOylation) modulate phase separation; (3) Discovering small molecules or peptides capable of regulating pathological condensates; and (4) Analyzing and elucidating aberrant condensate-chromatin binding interactions and their underlying regulatory mechanisms (Fig. 3).
